# The impact of different spectacle designs on the European visual field requirements for driving

**DOI:** 10.1016/j.optom.2025.100582

**Published:** 2025-10-13

**Authors:** Thea Melsen Sudmann, Anne Kathinka Jeber, Anne Brækhus, Ole Klungsøyr, Fiona J. Rowe, Øystein Kalsnes Jørstad

**Affiliations:** a Department of Ophthalmology, Oslo University Hospital, Oslo, Norway; b Institute of Clinical Medicine, Faculty of Medicine, University of Oslo, Oslo, Norway; c The Norwegian National Centre for Ageing and Health, Department of Neurology, Oslo, University Hospital, Oslo, Norway; d The Memory Clinic, Department of Geriatric Medicine, Oslo University Hospital, Oslo, Norway; e Oslo Centre for Biostatistics and Epidemiology, Department of Research and Innovation, Oslo University Hospital, Oslo, Norway; f Institute of Population Health, University of Liverpool, Liverpool, UK

**Keywords:** Visual field, Perimetry, European driving test, Spectacles, Visual field standards for driving

## Abstract

**Purpose:**

Spectacles can obstruct the peripheral visual field (VF) and interfere with formal requirements for driving. The European VF standards can be assessed with perimetry using the European Driving Test (EDT). This study aimed to evaluate the impact of different spectacles on the EDT, and their compliance with the European VF standards for driving.

**Methods:**

This cross-sectional study included 30 participants (15 males and 15 females) with normal VF. Participants underwent binocular EDT perimetry with three different spectacles. The number of missed test points were recorded and the vertex distance (VD), pupillary distance, and eye dominance were measured. Statistical comparisons were conducted using a generalized linear mixed model, with significance set at *p* < 0.05.

**Results:**

Peripheral VF loss was observed in 11 (37 %) participants (10 males) with spectacle B and in six (20 %) participants (five males) with spectacle C, whereas only one participant had a single missed test point with spectacle A. Participants with spectacle-related VF loss had significantly greater VD than those without. Moreover, there were a higher number of missed test points on the side of the dominant eye.

**Conclusions:**

Spectacles with thin frames and temples had a negligible impact on the peripheral VF, whereas thicker frames and temples could compromise compliance with the VF standards. VF loss was associated with greater VD, which can explain why male participants exhibited more artefacts. These findings emphasize the need to consider spectacle design in fitness-to-drive assessments with perimetry and raise awareness of potential VF restrictions associated with certain eyewear.

## Introduction

Spectacle frames can obstruct the peripheral visual field, with the extent of obstruction depending on the frame's physical characteristics and its positioning relative to the eyes.^1^^,^^2^ It has long been recommended that aviation pilots wear glasses with large lenses and thin frames to minimize obstruction of peripheral vision.^3^ Similarly, it is important to consider the potential negative impact of the frame and temples in a fitness-to-drive context. Avoiding spectacle frames that obstruct the visual field is crucial during testing, as artefacts can have two distinct implications: misdiagnosis—leading to unnecessary concern and diagnostic work-up —and the unwarranted revocation of driving privileges.

According to the European visual standards for driving, group-1 driving license (car and motorcycle) holders are required to have a binocular visual field of at least 120° horizontally (with a minimum of 50° to both the left and right) and 40° vertically (with a minimum of 20° upwards and downwards).^4^ Accurate assessment of the visual field should involve automated perimetry, ideally using the European Driving Test (EDT), as this perimetry algorithm specifically adheres to the European visual field criteria for driving.^5^

To diagnose visual field loss with perimetry, clinicians commonly use threshold programs that focus on the central 20–30° of the visual field, where spectacle frames have minimal influence.^6^ In contrast, EDT assesses the visual field up to 70° temporally, making it more susceptible to spectacle frame artefacts, which may influence the fitness-to-drive assessment. Understanding how different frames affect the visual field in this context is essential, as the European visual field standards have broad applicability across Europe, encompassing the 30 European Union and European Economic Area member states, as well as Switzerland and the United Kingdom. However, there remains a lack of knowledge regarding the impact of spectacle frame artefacts when using wide-field perimetry—such as the EDT—to evaluate compliance with the European standards.

The purpose of this study was to assess the impact of three different spectacles on the peripheral visual field, as measured with the EDT, and to evaluate their compliance with the European visual field criteria for driving.

## Methods

This cross-sectional study was conducted at the Department of Ophthalmology, Oslo University Hospital (OUH), with participant examinations taking place between October 20, 2024, and December 20, 2024. The study received approval from the Institutional Data Protection Officer at OUH (reference 24/18,562). The Regional Committee for Medical Research Ethics South-Eastern Norway also reviewed the study protocol and concluded that their approval was not required to conduct this study (reference 806,881).

Study candidates were recruited through information shared with colleagues, friends, and family. The inclusion criteria were: (I) binocular visual acuity of ≤ 0.00 logarithm of the minimum angle of resolution, either uncorrected or with contact lenses; (II) a normal binocular visual field in both eyes, either uncorrected or with contact lenses; (III) age between 18 and 40 years (to avoid presbyopia correction in the perimeter); and (IV) signed informed consent. Inclusion criteria (I) and (II) were assessed after obtaining informed consent. Visual acuity was measured using an ETDRS chart, and the binocular visual field was evaluated with the EDT, using an Octopus 900 perimeter (Haag-Streit, Köniz, Switzerland). A normal binocular visual field was defined as having an EDT result with no more than three scattered missed test points when tested without spectacles, along with no more than three false answers. If more than three false answers occurred, the EDT was repeated.

**Fig. 1:**
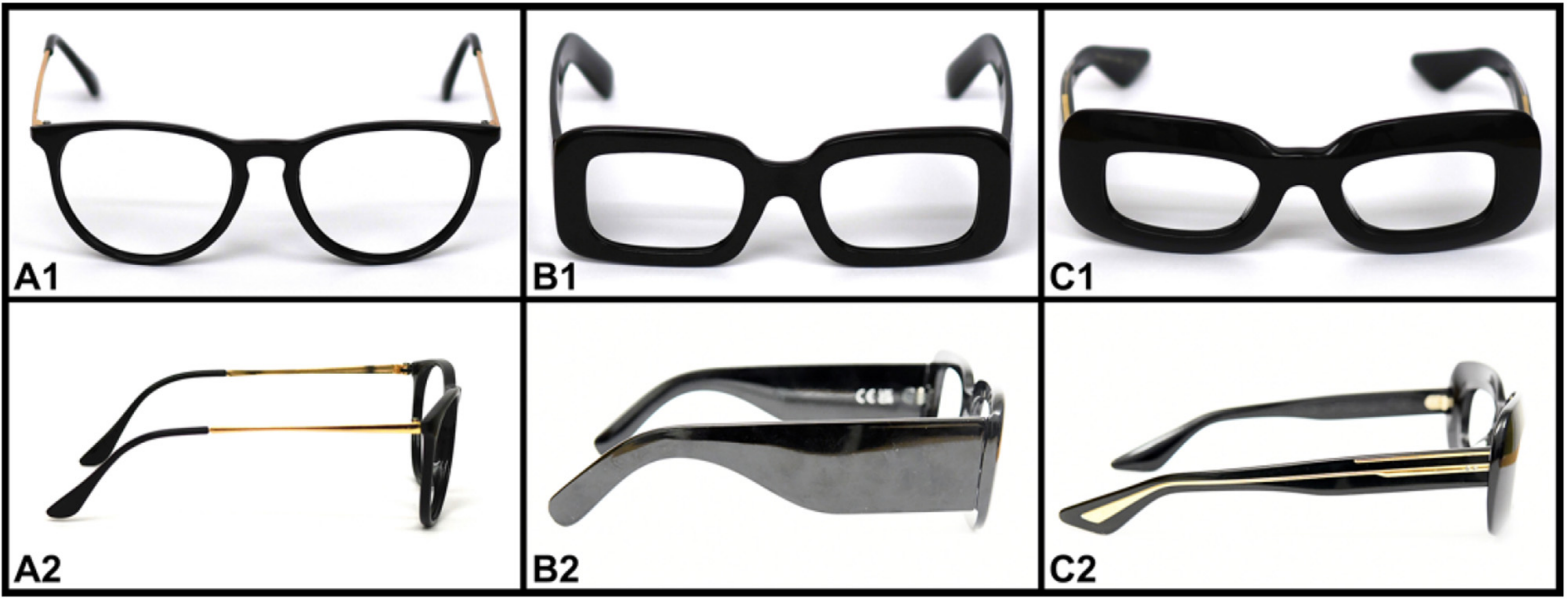
Front- and lateral-view pictures of the three spectacles (A, B, and C) from the study. Spectacle A has thin frames and temples, spectacle B has medium-thickness frames and thick temples, and spectacle C has thick frames and medium-thickness temples.

**Fig. 2:**
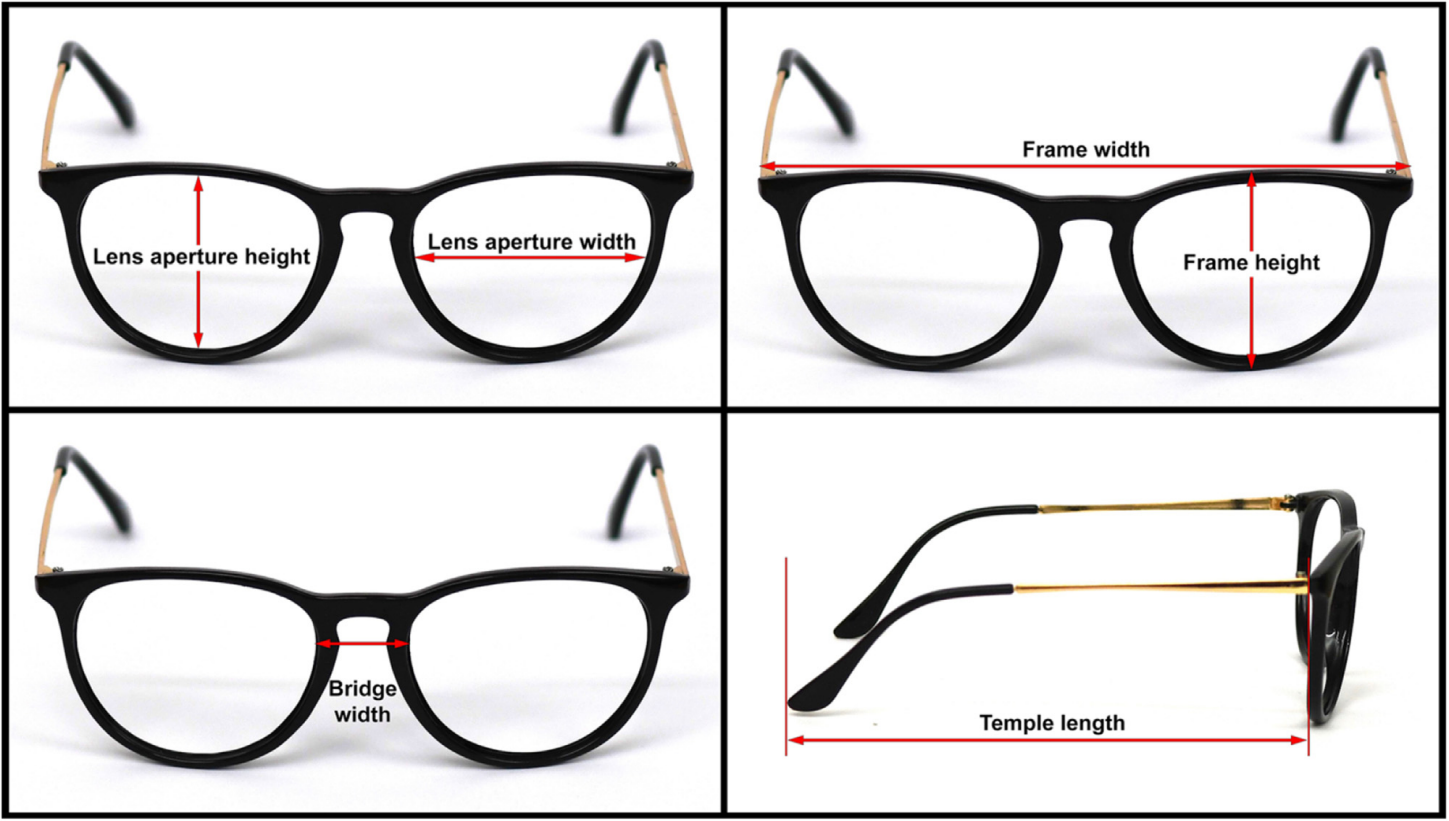
Spectacle frame measurements.

Participants who met the inclusion criteria underwent three additional perimetry examinations with the EDT, wearing Conformité Européenne (CE)-marked spectacles with (A) thin frames and temples, (B) medium-thickness frames and thick temples, or (C) thick frames and medium-thickness temples. To prevent lenses and refraction from interfering with the perimetry test result, we removed them from the frames prior to testing. The three examinations using different spectacles were performed in random order. Fig. 1 provides pictures of the three spectacles. Fig. 2 and Table 1 show the dimensions we measured. To prevent the lenses from interfering with the perimetry light stimuli, we removed them from the frames prior to testing. We measured each participant’s eye dominance using the Miles test^7^ and recorded their pupillary distance (PD) and vertex distance (VD) using a ruler. The participants put on their assigned randomized spectacles in a position they felt was comfortable. We then measured the VD as the distance from the closed eyelid to the inner margin of the upper part of the spectacle frame, subtracting one mm to account for eyelid thickness.

**Table 1:** Spectacle frame dimensions.

Measurement (in mm)	Spectacle A	Spectacle B	Spectacle C
Lens aperture height	4.5	3.5	2.8
Lens aperture width	5.1	4.7	4.7
Frame width	141.0	138.0	143.0
Frame height	49.0	39.0	28.0
Bridge width	20.0	23.0	24.0
Temple length	12.9	13.4	13.9
Temple width	0.4	3.0	1.2

To determine whether a visual field of 50° to the left or right meets the European visual field requirement of at least 120° horizontally, the EDT measures the visual field up to 70° temporally (50° + 70° = 120°). In accordance with this requirement, the best perimetry result was identified in the 120° horizontal range, which contains 112 test points. Mean and standard deviation (SD) number of missed test points were used to describe the EDT results quantitatively and Cohen’s d to determine whether the differences between the spectacles were practically meaningful. To account for dependency of results within the same individuals, a generalized linear mixed model and the Akaike Information Criterion for model selection were used. A power calculation using a paired *t*-test, with a two-sided significance level of 0.05 and 80 % power, indicated that 33 participants would be required to detect a medium effect size (Cohen’s *d* = 0.5). All calculations were performed using R version 4.3.3.

## Results

**Fig. 3:**
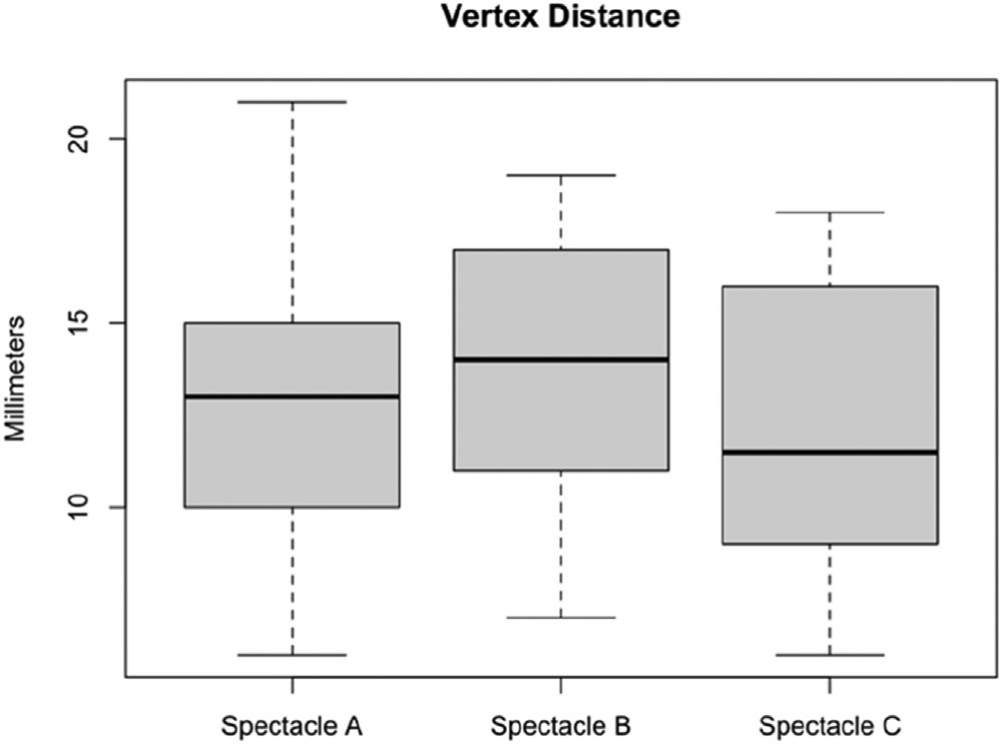
Boxplot of the participants’ vertex distance for each spectacle.

Thirty-two candidates provided signed informed consent and were screened for inclusion in the study. Two candidates did not meet the visual acuity criterion, bringing the final sample size to 30 participants, as planned. There were 15 male and 15 female participants, with a mean (SD) age of 32.1 (3.9) years and mean (SD) PD of 62.8 (3.9) mm. The mean (SD) number of missed test points from spectacle A, B, and C within the best 120° x 40° visual field was 0.0 (0.2), 1.6 (2.8), and 0.8 (2.2). The mean (SD) VD was 12.7 (3.9) mm when wearing spectacle A, 13.6 (3.5) mm when wearing spectacle B, and 12.3 (3.8) mm when wearing spectacle C. Fig. 3 presents boxplots of the participants’ VD for each spectacle. Male participants had higher mean VD than female participants when wearing spectacle A (15.3 mm versus 10.0 mm; *p* < 0.001), B (16.0 mm versus 11.3 mm; *p* < 0.001), and C (15.3 mm versus 9.3 mm; *p* < 0.001).

**Fig. 4:**
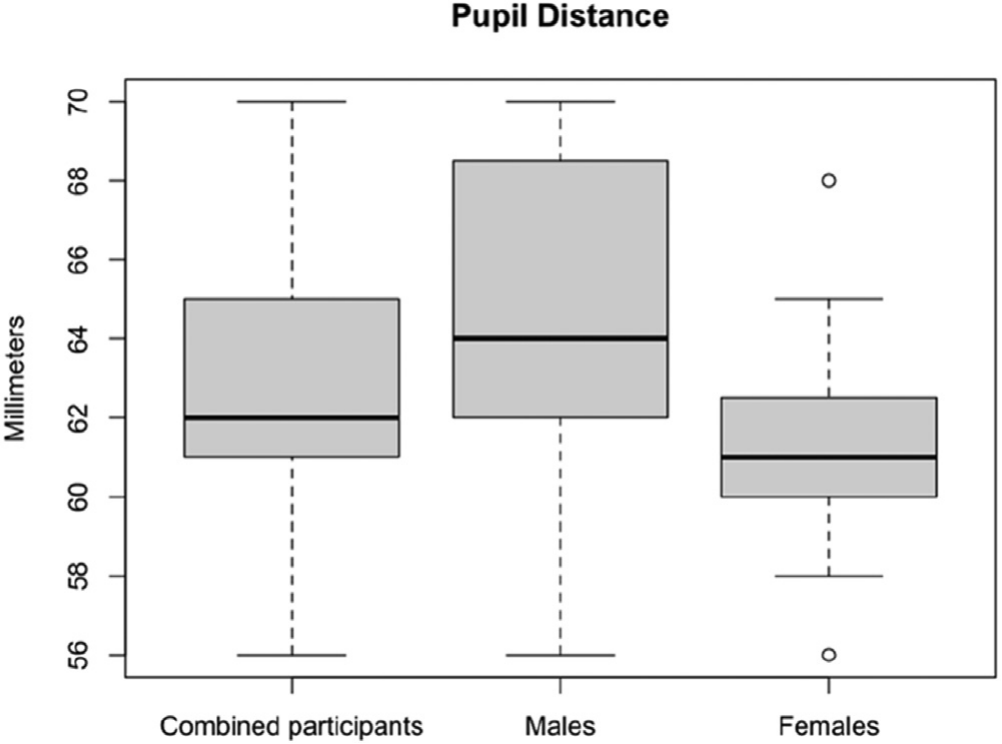
Boxplot of the pupil distance for male, female, and combined participants.

Male participants also had higher mean PD than female participants (64.3 mm versus 61.3 mm; *p* = 0.03). Fig. 4 presents boxplots of the PD for male, female, and combined participants.

Seven participants were left-eye dominant, while 23 were right-eye dominant.

Only one male participant demonstrated peripheral visual field loss while wearing spectacle A, affecting just a single test point. On the other hand, 11 (37 %) participants (one female and ten males), exhibited peripheral visual field loss with spectacle B, and six (20 %) participants (one female and five males) exhibited peripheral visual field loss with spectacle C, as indicated by missed test points. Out of the 112 test points in the best 120° × 40° visual field, the mean (SD) number of missed test points among the participants who exhibited peripheral visual field loss was 4.3 (3.1) for spectacle B and 3.8 (3.7) for spectacle C. Between spectacle A and B, Cohen’s d showed a medium to large effect (*d* = 0.79), between spectacle A and C it showed a medium effect (*d* = 0.48), and between spectacle B and C it showed a small to medium effect (*d* = 0.32).

A generalized linear mixed model was conducted, adjusting for the dependency of repeated measurements withing the same individuals. The random intercept showed significant variance (*p* < 0.01), indicating the contribution of the within-subject factor (dependence) in the model. The different spectacles, VD, and PD were incuded as variables in the generalized linear mixed model. Spectacle B caused significantly more visual field loss compared to spectacle A, with an odds ratio (OR) of 34.90 (95 % CI 1.96–620.00; *p* = 0.02). Spectacle C did not cause significantly more visual field loss compared to spectacle A, with an OR of 8.56 (95 % CI 0.80–92.20; p = 0.08). Visual field loss was associated with higher VD, with an OR 3.64 (95 % CI 1.08–12.20; p = 0.04). In contrast, PD had a non-significant effect, with an OR of 2.35 (95 % CI 0.68–8.17; p = 0.18). The most significant contributors to visual field loss were wearing Spectacle B and having a higher VD.

**Fig. 5:**
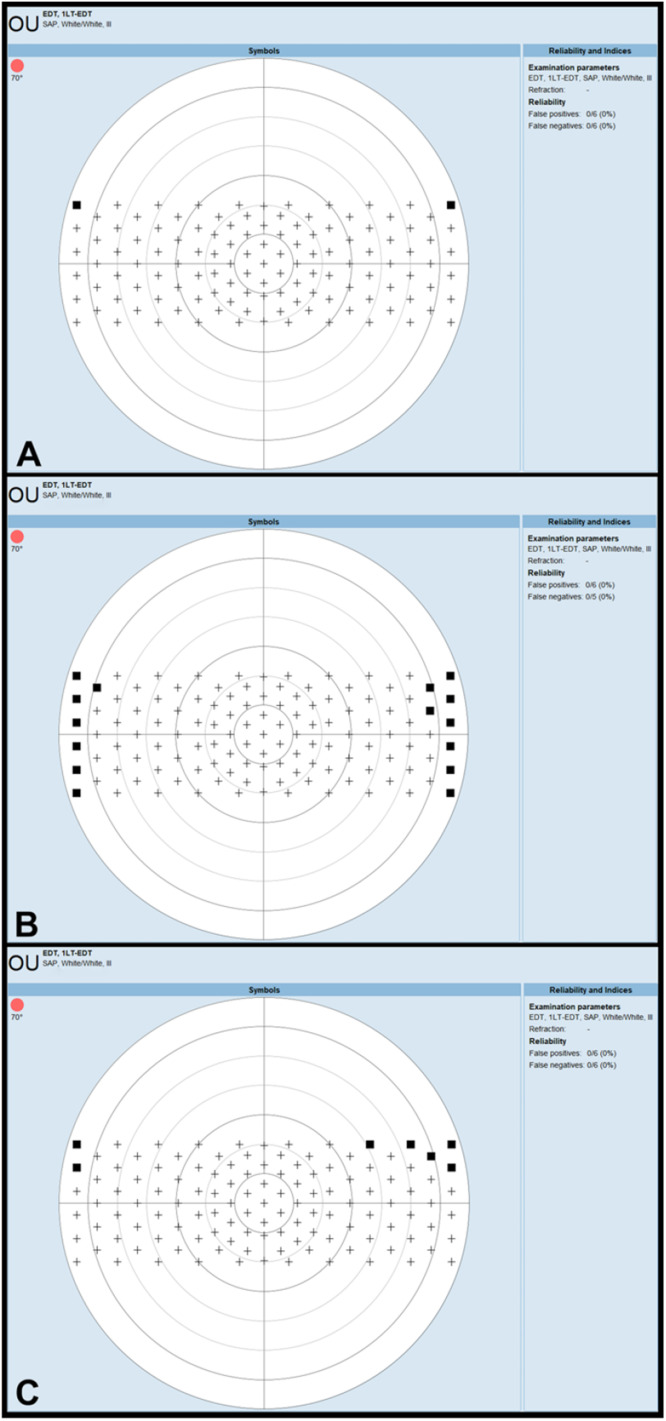
European Driving Test printouts of a study participant carried out with spectacle A (thin frames and temples), spectacle B (medium-thickness frames and thick temples), and spectacle C (thick frames and medium-thickness temples). The black squares indicate missed peripheral test points. Spectacle B and C both cause three missed test points in the best 120° x 40° visual field, which must be preserved according to the European visual field requirements for driving.

None of the participants demonstrated central visual field loss related to the spectacles. Fig. 5 shows the EDT results of a study participant.

As shown in Table 2, participants with spectacle-related peripheral visual field loss had greater mean PD and VD than participants with normal visual field, but the difference was only statistically significant for mean VD.

**Table 2:** Pupillary and vertex distance (in mm) of participants with and without spectacle-related peripheral visual field loss, as indicated by missed test points.

	Spectacle B		Spectacle C	
	Loss (*n* = 11)	No loss (*n* = 19)	Loss (*n* = 6)	No loss (*n* = 24)
Mean (standard deviation) vertex distance	15.6† (3.3)	12.5† (2.7)	14.8‡ (2.9)	11.7‡ (3.8)
Mean (standard deviation) pupillary distance	64.6 (4.4)	61.8 (3.3)	65.0 (3.3)	62.3 (3.9)

† Statistically significant difference between means (*p* = 0.02).

‡ Statistically significant difference between means (*p* = 0.047).

With regard to eye dominance of participants with peripheral visual field loss, we observed a higher number of missed test points on the side of the dominant eye (Table 3).

**Table 3:** Relationship between eye dominance and lateralization of peripheral visual field loss.

	Spectacle B				Spectacle C			
Eye dominance	Right eye		Left eye		Right eye		Left eye	
Hemifield	Right	Left	Right	Left	Right	Left	Right	Left
Mean (standard deviation) number of missed test points in the best 120° x 40°	5.8 (4.1)	3.1 (2.7)	1.8 (3.0)	3.3 (4.3)	2.2 (2.3)	2.1 (1.5)	3.3 (4.0)	5.0 (5.2)

Note that seven participants were left-eye dominant, while 23 were right-eye dominant.

## Discussion

Spectacle frames can obstruct the peripheral visual field, which is particularly relevant for driving, since formal visual standards must be met. In this study involving 30 healthy volunteers, we used the EDT perimetry algorithm to evaluate how three different spectacle frames affected the peripheral visual field and complied with the European visual field standards for driving. We found that a pair of spectacles with thin frame and temples had a negligible effect on the visual field, whereas two different spectacles with thicker frames and temples restricted the peripheral visual field in several participants. The distance of the spectacles from the eyes also influenced the visual field results, with participants experiencing spectacle frame-related visual field loss having a significantly greater VD. We conclude that spectacles with thin frames and temples can be worn during fitness-to-drive assessments with perimetry. In contrast, thicker frames and temples can induce spectacle frame artefacts, particularly when the spectacles are worn with a greater VD. Attention should be paid to the fact that some spectacles may be unsuitable for driving, potentially putting the driver at risk of failing to meet legal requirements.

As explained in the Introduction, the European visual standards for driving emphasize the temporal visual field, which must be at least 50° to both the left and right and at least 120° in total. The EDT assesses the visual field up to 70° temporally, making this region particularly susceptible to spectacle frame artefacts. Correspondingly, spectacle frame-related visual field loss typically appeared as peripheral temporal defects in our study. Moreover, spectacle B, which only had medium-thickness frames but thick temples, interfered more with the temporal visual field than spectacle C, which had thick frames but only medium-thickness temples. The wide confidence intervals from the generalized linear mixed models indicated considerable variability, with some participants showing no visual field loss and others showing substantial loss. Still, this finding indicates that temple width is a key spectacle property in preserving—or limiting—the temporal visual field necessary to comply with the European visual field standards for driving. On the other hand, the visual field standards only require a 20° visual field upwards and downwards, and this region was not susceptible to spectacle frame artefacts in our study—even with spectacle C, which had the thickest frames.

A strict interpretation of the European visual field requirements for driving suggests that no test points may be missed within the 120° × 40° field assessed by the EDT. In practice, however, some flexibility exists in interpreting perimetry results, although no standardized or universally accepted approach has been established across Europe. Instead, the enforcement of visual field standards varies between countries.^8^ Consequently, the legal implications of spectacle frame artefacts, as demonstrated in this study, will depend on the specific national context—having the greatest impact in countries with stricter interpretations of perimetry results.

Performing perimetry without spectacles may seem like a simple way to avoid spectacle frame artefacts during a fitness-to-drive assessment. However, it must be kept in mind that perimetry testing without proper refractive correction reduces the intensity of the light stimulus, with the extent of reduction also depending on the pupillary diameter—for example, a 1.10-dB decrease in averaged macular sensitivity per dioptre of refraction error for a 3-mm-in-diameter pupil.^9^ The Esterman program, which some countries endorse for assessing fitness to drive, presents a fixed 10-dB supra-threshold stimulus.^8^ This is a very bright stimulus, making the Esterman program relatively robust against uncorrected refraction errors if performed without glasses. In contrast, EDT presents a dynamic 8-dB supra-threshold stimulus that relates to the physiological hill of vision and decreases towards the central visual field.^5^ An uncorrected refractive error lowers this threshold, making it more difficult to pass the test. To ensure a fair fitness-to-drive assessment of the visual field, drivers with significant refraction errors should therefore wear their own spectacles during binocular EDT testing (perimeters typically provide only a single trial lens holder). Our study shows that wearing spectacles does not affect the peripheral visual field, if they have thin frames and temples.

The European visual standards for driving are one of thousands of legislative acts applicable within the European Economic Area.^4^ There are also European standards for prescription spectacles, which are classified under the Medical Device Regulation, and sunglasses, which are regulated under the Personal Protective Equipment Regulation.^10^ The CE mark is a certification indicating that a product complies with relevant European regulations and directives. All three spectacles in this study were CE-marked, but two of them could still induce spectacle frame artefacts that were incompatible with the European visual field standards for driving. The lack of specific European regulations for driving eyewear means that some spectacles, despite bearing the CE mark, may still interfere with a driver's visual field and jeopardize road safety.

Previous research has demonstrated a relatively weak correlation between visual field loss and driving hazards, and we anticipate that establishing a direct link between spectacle frame obstruction and driving risk would likewise be challenging.^11^, ^12^, ^13^ Nevertheless, we believe an optimal, unobstructed visual field should be prioritized when driving, regardless of whether spectacle-induced obstruction can be conclusively shown to pose a hazard.

Unlike Europe, the State of California prohibits driving while wearing glasses with temples that are at least half of an inch (1.27 cm) if they extend below the horizontal centre of the lenses and obstruct lateral vision.^14^ This law applies to the temple width of spectacle B in our study, effectively banning it for driving in California. However, our study shows that other factors, such as frame thickness and the positioning of spectacles relative to the eyes, can also interfere with the peripheral visual field, and more comprehensive regulation would be necessary to ensure adequate visual field coverage. While regulating driving eyewear in Europe may not be a practical solution, raising awareness about the impact of spectacle frame design on peripheral vision remains crucial. We suggest the implementation of public information campaigns to raise awareness about to impact of spectacle obstruction during driving. This issue is particularly important for both drivers and retailers, as informed choices about driving spectacles can help minimize visual obstructions and ensure overall safety on the road. Additionally, we recommend the use of thin-frame spectacles with presbyopic correction (if necessary) during binocular perimetry as part of fitness-to-drive assessments.

This study has several limitations. First, the Octopus 900 perimeter is primarily designed for monocular testing, with a bi-curved chin support that centres the tested eye. During binocular perimetry, as in this study, the dominant eye is centred by placing the chin on the left chin support for right-eye dominance and on the right chin support for left-eye dominance. This induces a one-PD lateral displacement of the non-dominant eye, which, in the 30-cm-radius half-dome of the perimeter, reduces the test angle for the EDT peripheral visual field test from 70° to about 65° on the side of the non-dominant eye. In other words, centring the dominant eye during binocular perimetry testing introduces a bias, resulting in a somewhat more lenient test on the side of the non-dominant eye, as evidenced by a higher number of missed test points on the side of the dominant eye in our study. Second, we only tested three spectacles in this study, while there are thousands of spectacles with different designs available. Moreover, all participants wore the same spectacles, regardless of individual differences in fit. Broader and more prominent facial features likely contributed to the greater VD in male participants, and, considering the correlation between VD and visual field loss, this likely explains why visual field loss was more frequently observed in male participants. To increase ecological validity in future studies, more spectacle options could be included, with frames selected to best fit each participant, as would occur in a retail setting. Furthermore, age- and race-dependent facial characteristics may influence the positioning of the spectacles relative to the eyes, potentially affecting the visual field results. In this regard, recruiting participants from friends, family, and colleagues—restricted to those under 40 years of age and within a Norwegian setting—introduces sampling bias, as anatomical facial structures are limited to younger individuals, primarily of Caucasian descent. The study was also slightly underpowered, with only 30 participants included, whereas the power analysis indicated that 33 participants were needed. Third, while beyond the scope of our study, it is important to note that not only spectacle frames but also lenses may affect the visual field.^6^ Lenses with high corrective power can distort the peripheral visual field, and filters can reduce light intensity, potentially amplifying visual field loss caused by spectacle frames. Finally, we measured spectacle frame artefacts in eyes that were centred during perimetry. In contrast, drivers actively move their eyes to gather visual information about their surroundings (visual scanning), which increases the visual obstruction caused by a pair of spectacles on the same side as the gaze direction. Consequently, spectacle frames and temples may intermittently obstruct the peripheral visual field even more during driving than was demonstrated in our study.

In conclusion, spectacle frames and temples can obstruct the peripheral visual field, particularly in the temporal region, which is crucial for meeting European visual field driving standards. While thin frames and temples have minimal impact, thicker designs can cause significant visual field restrictions, highlighting the need for awareness when performing fitness-to-drive assessments with perimetry or selecting eyewear for driving.

## Funding information

This research project received funding from The Norwegian Association of the Blind, the Norwegian Glaucoma Research Foundation, Arthur and Odd Clauson's legacy, and Oslo University Hospital.

## Declaration of competing interest

FR receives consultancy funding from Haag-Streit AG. TMS, AKJ, AB, OK, and ØKJ have no conflict of interest to disclose.
